# Optimized Carbon–Nitrogen Fertilization Boosts Fragrant Rice (*Oryza sativa* L.) Yield and Quality via Enhanced Photosynthesis, Antioxidant Defense, and Osmoregulation

**DOI:** 10.3390/plants14121832

**Published:** 2025-06-14

**Authors:** Wenjun Xie, Zhe Jiang, Li Lin, Xinyi Wang, Lihe Zhang, Zhaowen Mo

**Affiliations:** 1 State Key Laboratory for Conservation and Utilization of Subtropical Agro-Bioresources, College of Agriculture, South China Agricultural University, Guangzhou 510642, China; wenjunxie52@163.com (W.X.); jiangzscau@foxmail.com (Z.J.);; 2 Engineering Research Center of Biotechnology for Active Substances, Ministry of Education, Chongqing Normal University, Chongqing 401331, China

**Keywords:** fragrant rice, carbon–nitrogen fertilizer, yield, grain quality, physiological response

## Abstract

The effects of the foliar spraying of carbon and nitrogen on the yield and quality of fragrant rice (*Oryza sativa* L.) remain unknown. A two-year field experiment was conducted by using two fragrant rice varieties, Meixiangzhan 2 and Xiangyaxiangzhan, as experimental materials. Three carbon treatments (C0: 0 mg L^−1^ glucose, C1: 150 mg L^−1^ glucose, and C2: 300 mg L^−1^ glucose, recorded as C0, C1, and C2, respectively) and three nitrogen treatments (N0: 0 mg L^−1^ urea, N1: 50 mg L^−1^ urea, and N2: 100 mg L^−1^ urea) were employed as experimental treatments, and there were a total of nine treatments (C0N0, C0N1, C0N2, C1N0, C1N1, C1N2, C2N0, C2N1, and C2N2). The yield and quality of fragrant rice were investigated. The results show that carbon and nitrogen treatments significantly affected the yield and grain number per panicle in fragrant rice. The yields of the two cultivars under C1N2 and C2N1 were maintained at high levels. This was mainly because the C1N2 and C2N1 treatments resulted in higher grain number per panicle and grain filling percentage. Carbon and nitrogen treatments significantly increased the dry weight and photosynthesis parameters of fragrant rice. The carbon and nitrogen treatments significantly increased the protein content. The improved grain yield was related to improvements in photosynthesis and antioxidant defense as well as osmoregulation. In summary, optimized spraying of 150 mg L^−1^ glucose and 100 mg L^−1^ urea at the booting stage benefits yield and grain quality by regulating photosynthesis, antioxidant defense, and osmoregulation.

## 1. Introduction

Rice (*Oryza sativa* L.) is one of the major food crops in China, with 65% of the population consuming rice as a staple food [1]. With improvements in living standards, the demand for high-quality rice from consumers is increasing [2]. Fragrant rice stands out among high-quality rice because of its unique aroma and is favored by consumers [3]. The demand for fragrant rice is high, yet its supply is relatively low, and it cannot meet existing market demand, thus exacerbating the conflict between supply and demand [4,5]. The key to solving this shortage problem is to improve the yield and quality. Studies have shown that these can be significantly increased through the implementation of scientific cultivation and management measures, including precise fertilization [6,7], optimization of the planting method [8,9], and rational regulation of the water supply [10,11], which provide strong support for the high-yield and high-quality cultivation of fragrant rice.

Nitrogen is an important source of nutrients for rice growth and is decisive for the photosynthetic characteristics, yield, and quality of rice [12]. Increased nitrogen application can effectively promote rice yield formation [13] and has been found to improve the appearance, quality, retrogradation rate, proportion of medium-sized starch particles, and relative crystallinity of the rice grains [14]. However, rice has an absorption limit for nitrogen fertilizer. Beyond a certain range, its nitrogen absorption and metabolism efficiency decline [15]. Moreover, the excessive application of nitrogen fertilizer affects the sustainability of the planet [16]. Research has demonstrated that optimized fertilizer management enhances nitrogen use efficiency (NUE), reducing application rates while maintaining high yields [17]. It has also been shown that balanced nitrogen reduction and partial nitrogen reduction in basal fertilizers can increase the variety of rice processed, reduce chalkiness, and improve texture while maintaining high yields [18]. Changing the application rate of nitrogen at various growth stages of rice can improve the source–sink contradiction in yield formation and further increase rice yield [19]. Zhao et al. [20] showed that exogenous application of glucose or sucrose could promote the photosynthetic capacity and carbon assimilation capacity of maize. Some studies have shown that spraying sugars can increase the photosynthetic rate and nitrogen metabolism efficiency, which is beneficial to the metabolism and uptake of nitrogen fertilizer in rice, implying that sugars and nitrogen fertilizer can synergistically promote the growth of rice [21]. Therefore, it is important to study the effects of carbon and nitrogen interaction treatments on the yield and quality of fragrant rice to increase the maximum amount of nitrogen fertilizer applied and promote the yield and quality of rice.

Sucrose produced by photosynthesis is the most common carbohydrate in plants. A portion of it is used to support growth and development, and the rest is stored as starch in the absorption pool [22]. Excess photosynthesis products are stored as transitional starch in light source tissues during light periods and are reused for growth, development, or storage during dark periods [23]. Therefore, improving the transfer of carbohydrates and increasing the accumulation of carbohydrates may be one of the main ways to increase rice yield [24]. At the molecular level, the limitation of starch synthesis through ADP-glucose pyrophosphorylase can be alleviated by adjusting key enzymes in the carbohydrate synthesis and transfer pathway to promote photosynthesis-induced yield increases in crops [25,26]. Research indicates that optimized agronomic practices, such as moderate application of carbon or nitrogen supplements, enhance carbon–nitrogen accumulation and metabolism, promoting efficient starch translocation to grains for improved yield and quality. Optimal glucose application also stimulates chlorophyll biosynthesis in crops [27] and simultaneously stimulates the activities of Ribulose-1,5-bisphosphate carboxylase/oxygenase (Rubisco) [28] and β-carotene production-related enzymes [29]. Ma et al. [30] showed that spraying a certain concentration of glucose changed the selection of nitrogen sources by the root system, promoting the uptake of organic nitrogen and reducing the uptake of nitrate. Jiang et al. [31] reported that the application of sucrose spray could enhance the efficiency with which photosynthetic products are transported to spikes, which may lead to higher grain yields. Lyu et al. [32] reported that nitrogen fertilizer application can increase the carbon and nitrogen metabolism ability of rice, thus achieving simultaneous improvements in rice yield and quality. Guo et al. [33] reported that exogenous carbon and nitrogen treatments promoted the intensity of photosynthesis and the transfer of storage material to the spikes in spring wheat, resulting in a decrease in the proportion of ineffective spikes, which ultimately induced an increase in the number of grains in each spike and the thousand grain weight. Zhang et al. [34] reported that the simultaneous application of carbon and nitrogen fertilizers could reduce the protein content and improve the taste of rice.

Nitrogen and carbon are the core elements for rice growth. Nitrogen combines the functions of morphology construction, signal regulation, and yield formation. It is absorbed by the root system and reacts enzymatically to produce nitrogenous compounds, such as amino acids, which are distributed to all parts of the plant by the translocation system to maintain growth and reproduction [35]. Research by Wu et al. [36] indicates that nitrogen can regulate the number of tillers in rice by interacting with gibberellins. During the heading stage of rice, there is intense competition between the growth of the flag leaf and the formation of the panicle [37]. Timely supplementation of exogenous nitrogen, combined with sufficient endogenous nitrogen, can help rice plants acquire adequate nutrients during this period, reducing the number of degenerated spikelets and increasing the final filled-grain percentage [38], while also enhancing the storage protein content in the milled rice [39]. Photosynthesis is a crucial source of energy and carbon skeletons necessary for plant growth, with photosynthetic products primarily distributed in the form of sucrose through the phloem from the source (leaves) to different organs [40]. Green plants fix CO_2_ through photosynthesis to generate ribulose diphosphate, which is catalyzed by aldolase to form fructose-1,6-bisphosphate, and then synthesize sucrose through the cascade reaction of sucrose phosphate synthase and sucrose phosphate phosphorylase, completing the long-range transport of carbon [41,42]. Meanwhile, phosphoenolpyruvate produced by glycolysis is catalyzed by phosphoenolpyruvate carboxylase and pyruvate kinase to generate oxaloacetate and pyruvate, which enter the TCA cycle to provide energy and carbon skeletons for plant growth [43]. As a core pathway of cellular metabolism, nitrogen metabolism intersects with carbon metabolism in the TCA cycle, regulating and influencing various stages of plant growth and reproduction [44]. Insufficient nitrogen storage in plants can severely reduce the efficiency of photosynthetic carbon fixation [45]. Therefore, the balance and interaction between carbon metabolism and nitrogen metabolism are crucial for crop growth, development, and yield formation.

The effects of the simultaneous spraying of carbon and nitrogen substances have been verified in other crops, and the carbon and nitrogen effect in the regulation of the fragrance formation was investigated in our previous study [46]. However, few studies have reported the effects of spraying carbon and nitrogen substances on the yield and quality formation of fragrant rice. Therefore, in this study, the effects of carbon and nitrogen treatments on the yield, quality, dry matter, leaf photosynthesis, antioxidant defense, and osmoregulation of fragrant rice were investigated via synergistic treatment with different levels of exogenous glucose and urea. The aims of this study were to identify cultivation measures to achieve a high yield and superior quality of fragrant rice and to provide a theoretical basis for the cultivation of double-season fragrant rice in South China.

## 2. Materials and Methods

### 2.1. Experimental Description

**Figure 1:**
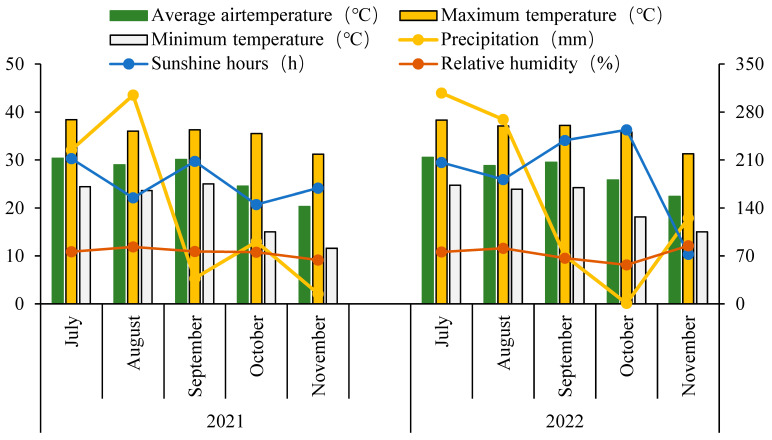
Mean monthly air temperature, maximum temperature, minimum temperature, precipitation, sunshine hours, and relative humidity during the rice (*Oryza sativa* L.) growing seasons of 2021 and 2022.

A two-year field trial was conducted from July to November in 2021 and 2022 at the Experimental Farm of the College of Agriculture (113°37′ E, 23°17′ N), South China Agricultural University, Guangzhou, China. The experimental region has a warm subtropical climate, and the soil during the study period was Acrisol. During the two-year experimental cycle, there were large differences in sunshine duration and precipitation from July to November, but there was little difference in the average air temperature, maximum temperature, minimum temperature, and relative humidity, with average temperatures ranging from 20 to 32 degrees in every month (Figure 1, data source: Climate and Agro-meteorological Center of Guangzhou). In 2021, the soil in the field trial had a pH of 6.22, and the organic matter, total N, total P, and total K contents were 22.41 g kg^−1^, 1.82 g kg^−1^, 0.84 g kg^−1^, and 10.32 g kg^−1^, respectively. The effective nitrogen was 244.99 mg kg^−1^, the effective phosphorus was 25.45 mg kg^−1^, and the effective potassium was 28.33 mg kg^−1^. However, in 2022, the soil pH was 6.61, and the organic matter, total N, total P, and total K contents were 28.37 g kg^−1^, 1.79 g kg^−1^, 0.83 g kg^−1^, and 10.06 g kg^−1^, respectively. The effective nitrogen was 240.59 mg kg^−1^, the effective phosphorus was 21.14 mg kg^−1^, and the effective potassium was 27.72 mg kg^−1^.

### 2.2. Experimental Design

Two fragrant rice cultivars, Meixiangzhan 2 and Xiangyaxiangzhan, which were provided by the College of Agriculture, South China Agricultural University, were used in this study. The experimental plots were set up with three different glucose treatments at concentrations of 0, 150, and 300 mg L^−1^, equivalent to application rates of 0, 150, and 300 g hm^−2^ glucose, respectively (denoted as C0, C1, and C2). Three urea treatments were applied at concentrations of 0, 50, and 100 mg·L^−1^, equivalent to application rates of 0, 50, and 100 g hm^−2^ urea, respectively (denoted as N0, N1, and N2). The plants in the experimental groups were sprayed during the booting stage, with full and uniform leaf spraying after 4 p.m. on a sunny and rainless afternoon, with no precipitation for 4 h after spraying [46]. A uniform spray volume of 100 mL m^−2^ was applied for each treatment to ensure complete and even leaf wetting.

The experimental plots were 25 m^2^ in size, and the planting density was 33 cm × 14 cm. Both cultivars were sown on 13 July 2021 and 14 July 2022, transplanted on 1 August 2021 and 3 August 2022, and harvested on 6 November in 2021 and 2022. A basal fertilizer of 90 kg hm^−2^ P_2_O_5_ and 150 kg hm^−2^ K_2_O, which was the only source of phosphate and potash, was applied 7 days before transplanting or sowing. Water and fertilizer management and weed, pest, and disease control were carried out according to local rice management practices.

### 2.3. Sampling and Measurements

#### 2.3.1. Measurement of Yield and Yield Components

At the maturity stage, 1 m^2^ of each plot was randomly selected for harvesting, and the number of panicles per m^2^ was investigated and replicated four times. The harvested grains were dried to a moisture content of 13.5 ± 1%, after which the grain yield, grain number per panicle, filled-grain percentage, and 1000-grain weight were measured [1].

#### 2.3.2. Measurement of Rice Quality

Full grains were selected from each treatment to determine the brown rice rate, milled rice rate, and head rice rate, based on the methods described by Mo et al. [47] and Gui et al. [1]. The protein content and amylose content were measured with a Near-Infrared Grain Analyzer (INFRATEC-1241, FOSS Corporation, Hillerød, Denmark). The chalky rice rate and chalkiness were measured with a Rice Appearance Quality Analyzer (SC-E, Hangzhou Wanshen Corporation, Hangzhou, China) [8].

#### 2.3.3. Measurement of Dry Weight and Leaf Area Index

Sampling was conducted from six representative plants from each plot at the heading and maturity stages; then, the leaf area was investigated at the heading stage, and the dry weight was measured at the maturity stage [9].

#### 2.3.4. Measurement of Photosynthesis Rate and Total Chlorophyll Content

At the heading stage, the photosynthesis rate of the flag leaves was measured via the portable photosynthesis system LI-6400 (LI-COR, Bourne, MA, USA) from 9:00 to 11:00 a.m. on sunny days according to the method described by [9].

The flag leaves of representative fragrant rice plants were selected from each plot at the flush stage, 15 days after the flush stage, and at the maturity stage, and the chlorophyll content was determined via a microtiter plate spectrophotometer according to the methods of Zhou et al. [48] and Li et al. [49].

#### 2.3.5. Measurement of Non-Structural Carbohydrate Content

The non-structural carbohydrate content was calculated by measuring the sugar and starch contents using biochemical kits (Boxbio Technology Co., Ltd., Beijing, China). The non-structural carbohydrate content was expressed as mg g^−1^ DW [46].

#### 2.3.6. Measurement of Antioxidant Defense, and Osmoregulation Parameters

At the heading stage, 15 days after heading, and maturity stage of fragrant rice, 20–30 flag leaves were collected from each plot. The leaves were immersed in liquid nitrogen for 30 s and stored at −80 °C in a freezer for subsequent determination of the antioxidant defense parameters, such as superoxide dismutase (SOD) activity, peroxidase (POD) activity, and catalase (CAT) activity, and the osmoregulation parameters, such as hydrogen peroxide (H_2_O_2_) content, malondialdehyde (MDA) content, ascorbic acid (AsA) content, and soluble protein content.

The SOD activity was measured using the nitroblue tetrazolium (NBT) method, with enzyme activity expressed as U mg^−1^ FW [49]. The POD activity was determined using the guaiacol method following Li et al. [49], with activity units as U min^−1^g^−1^ FW. The CAT activity was assayed using the ammonium molybdate method based on Pan et al. [50], with units as mmol min^−1^g^−1^ FW.

The MDA content was quantified using the method described by Li et al. [49] by measuring absorbance at 450 nm, 532 nm, and 600 nm, and expressed as μmol g^−1^ FW. The AsA content was determined according to Jiang et al. [51] by measuring absorbance at 534 nm, with results expressed in units of μg g^−1^ FW. The H_2_O_2_ content was measured following Lin et al. [52], with results expressed in units of nmol g^−1^ FW.

### 2.4. Statistical Analysis

The data of the experiment were processed using Microsoft Excel 2013, and multiple comparisons (least significant difference, LSD) were performed using Statistix 8.0 (Tallahassee, FL, USA). Before the comparisons, the data were tested for normal distribution using the “Shapiro–Wilk Normality Test”. A significant difference (*p* < 0.05, LSD) between treatments of the same cultivar is indicated by different lowercase letters. Graphing was performed using Microsoft Excel 2013. To investigate the correlations between variables, we performed correlation analysis in RStudio using R (version 4.3.1) and generated correlation heatmaps. To evaluate the correlations between yield, total dry weight, and grain quality data and the photosynthesis, antioxidant defense, and osmoregulation indicator data, we employed the Mantel test. The Mantel test assesses their relationships by calculating the correlation coefficient (Mantel’s r) and significance level (*p*-value) between two data groups. We visualized the correlations among yield, total dry weight, and grain quality data using the quickcor function from the ggcor package to generate corresponding heatmaps.

## 3. Results

### 3.1. Grain Yield, Total Dry Weight, and Yield Components

The grain yield and yield components were significantly different for the experimental years, perhaps due to the differences between the various experimental years (Figure 1, Table 1 and Table 2). The grain yield ranged from 3.57 t hm^−2^ to 6.07 t hm^−2^ and from 5.26 t hm^−2^ to 7.22 t hm^−2^ for Meixiangzhan 2 in 2021 and 2022, respectively. It ranged from 4.30 t hm^−2^ to 4.82 t hm^−2^ and from 4.24 t hm^−2^ to 6.58 t hm^−2^ for Xiangyaxiangzhan in 2021 and 2022, respectively. The carbon and nitrogen application treatments significantly affected the grain yield and total dry weight. A higher grain yield and total dry weight were detected for the carbon and nitrogen application treatments as compared to the C0N0 treatment. The C1N2 and C2N1 treatments represent the optimized carbon and nitrogen application combinations. Significant differences in grain yield and total dry weight were detected for Meixiangzhan 2 and Xiangyaxiangzhan. The cultivars’ interaction with the carbon and nitrogen application treatments significantly affected the total dry weight (Table 1).

The carbon and nitrogen application treatments significantly affected the grain number per panicle. Compared with the C0N0 treatment, the C0N1 and C1N2 treatments resulted in a significantly higher grain number per panicle in Xiangyaxiangzhan in 2022. The cultivar varied in terms of the filled-grain percentage and 1000-grain weight. Compared with the C0N0 treatment, the C1N2 treatment resulted in a reduction in the filled-grain percentage in Meixiangzhan 2 and an increase in the filled-grain percentage in Xiangyaxiangzhan in 2021. Compared with the C0N0 treatment, the C2N2 treatment resulted in a reduction in the filled-grain percentage in Meixiangzhan 2 in 2021 and 2022. Compared with the C0N0 treatment, the C2N0 treatment resulted in an improved 1000-grain weight in Meixiangzhan 2 in 2021, whereas the C1N0 and C2N1 treatments caused a decrease in the 1000-grain weight in Meixiangzhan 2 in 2022. The interaction between the cultivar and the carbon and nitrogen application treatments significantly affected the filled-grain percentage (Table 2).

### 3.2. Grain Quality

The different experimental years (Y) resulted in significant variation in the protein content, amylose content, chalky rice rate, and chalkiness. The cultivar (C) led to significant changes in grain milling quality, amylose content, chalky rice rate, and chalkiness. The carbon and nitrogen application treatments (T) significantly affected the protein content. The protein content and amylose content were significantly affected by the Y × C, Y × T, C × T, and Y × C × T. In 2021, for Xiangyaxiangzhan, the milled rice rate and head rice rate were highest in the C1N2 treatment, increasing by 3.81% and 9.37%, respectively, compared with those in the C0N0 treatment, whereas the milled rice rate and head rice rate were significantly lower in the C1N0 treatment. In 2022, for Meixiangzhan 2, the brown rice rate in the C1N1 treatment significantly increased by 12.57% compared with that in the C0N0 treatment, whereas that in the C0N2 treatment significantly decreased. Compared with the C0N0 treatment, the C0N1 and C2N1 treatments resulted in a greater brown rice rate, whereas the C1N0 treatment resulted in a significantly lower brown rice rate and milled rice rate. Compared with the C0N0 treatment, the C2N2 treatment resulted in a significantly greater head rice rate, whereas the C0N2 treatment resulted in a significantly lower percentage. In 2021, for Meixiangzhan 2, the protein content increased in all the treatments except C2N0 compared with that in the C0N0 treatment. Compared with the C0N0 treatment, the C2N0 and C2N1 treatments resulted in a significantly greater amylose content, whereas the C2N2 treatment resulted in a lower content. Compared with the C0N0 treatment, the C0N1 and C0N2 treatments resulted in significantly greater chalkiness. For Xiangyaxiangzhan, the protein content was significantly higher in C1N1 and C1N2, but lower in C2N1 and C2N2, compared to C0N0. The amylose content significantly increased in all the treatments compared with C0N0. In 2022, for Meixiangzhan 2, the protein content was greater in the C1N2 treatment and lower in the C2N2 treatment than in the C0N0 treatment. The C1N1 treatment resulted in the lowest chalky rice rate and chalkiness. Compared with those under the other treatments, the protein contents under the C1N1, C1N2, and C2N1 treatments significantly increased, whereas those under the C1N0, C2N0, and C2N2 treatments were significantly lower than those under the C0N0 treatment. Overall, the protein content, chalky rice rate, and chalkiness were greater under the C1N2 treatment than under the other treatments. However, the protein content, amylose content, chalkiness, and chalky rice rate were lower in the C2N2 treatment than in the C0N0 treatment (Table 3).

### 3.3. Photosynthesis Parameters

**Figure 2:**
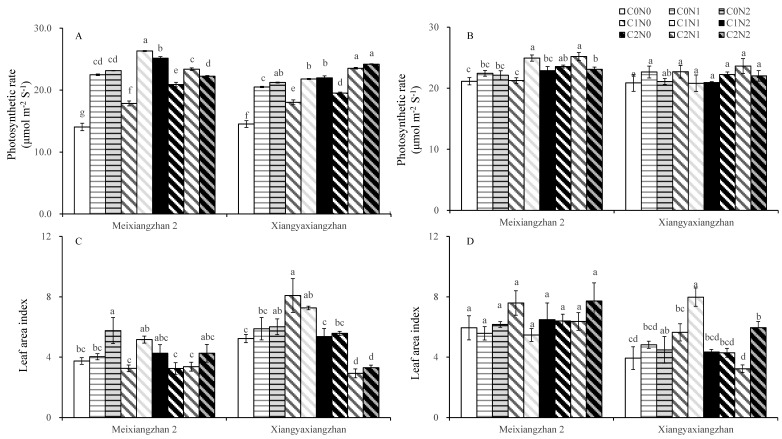
Effect of carbon and nitrogen fertilization at the booting stage on photosynthetic rate and leaf area index in fragrant rice at the heading stage. Different letters represent significant differences between treatments within a variety. (**A**,**B**): photosynthetic rate in 2021 and 2022; (**C**,**D**): leaf area index in 2021 and 2022. For a description of the treatments, see Table 1.

The carbon and nitrogen treatments improved the photosynthetic rate in flag leaves in fragrant rice at the heading stage. Compared with the C0N0 treatment, the photosynthetic rate for the carbon and nitrogen treatments was significantly improved in Meixiangzhan 2 and Xiangyaxiangzhan in 2021. A significant increment in the photosynthetic rate was detected for the C1N1, C2N0, C1N1, and C2N2 treatments for Meixiangzhan 2 in 2022, as compared to the C0N0 treatment (Figure 2A,B). Compared with the C0N0 treatment, the C0N2 treatment resulted in a significantly higher leaf area index in Meixiangzhan 2 in 2021. The C1N0 and C1N1 treatments significantly improved the leaf area index in Xiangyaxiangzhan in 2021 as compared to the C0N0 treatment, while the C2N1 and C2N2 treatments resulted in a lower leaf area index. The C1N1 and C2N2 treatments significantly enhanced the leaf area index in Xiangyaxiangzhan in 2022 (Figure 2C,D).

**Figure 3:**
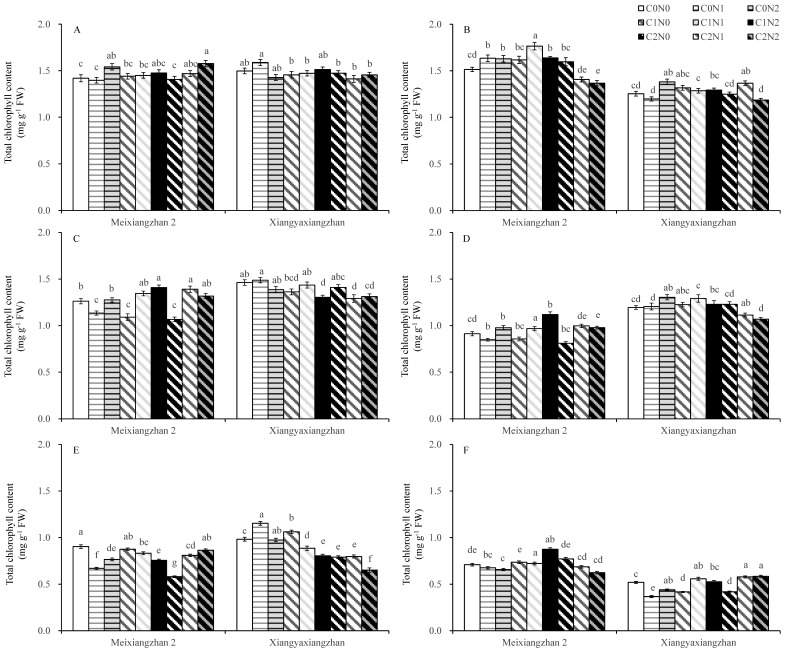
Effect of carbon and nitrogen fertilization at the booting stage on total chlorophyll content in fragrant rice at different stages. (**A**,**B**): heading stage in 2021 and 2022; (**C**,**D**): 15 days after heading in 2021 and 2022; (**E**,**F**): maturity stage in 2021 and 2022. Different letters represent significant differences between treatments within a variety. FW: fresh weight. For a description of the treatments, see Table 1.

The total chlorophyll content in fragrant rice varied significantly with the carbon and nitrogen treatments, growth stages, cultivars, and years. In 2021, for Meixiangzhan 2, the total chlorophyll content of fragrant rice increased under the carbon and nitrogen interaction treatments compared with the C0N0 treatment at the heading stage and for 15 days after the heading stage but decreased at the maturity stage. Compared with the C0N0 treatment, the C0N1 treatment improved the total chlorophyll content of Xiangyaxiangzhan at heading, 15 days after heading, and maturity. In 2022, for Meixiangzhan 2, the total chlorophyll content increased under the C1N1 and C1N2 treatments compared with the C0N0 treatment at the heading stage, 15 days after heading, and maturity stage, whereas for Xiangyaxiangzhan, the total chlorophyll content increased under the C1N1 and C1N2 treatments compared with the C0N0 treatment at the heading, 15 days after heading, and maturity stages, respectively (Figure 3).

### 3.4. Non-Structural Carbohydrate Content and Soluble Protein Content

**Figure 4:**
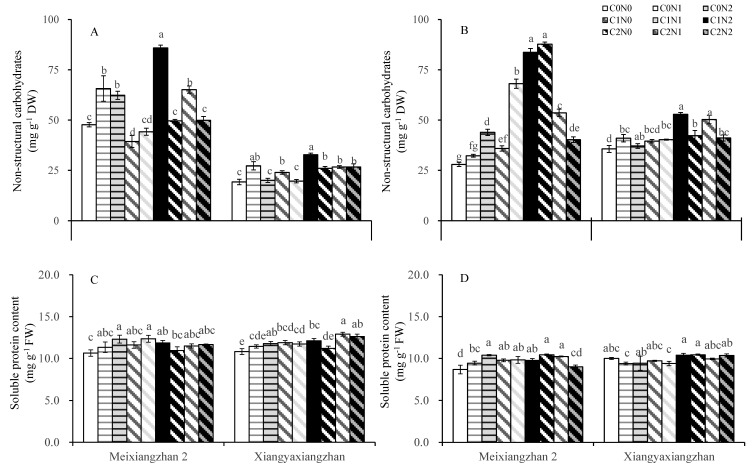
Effect of carbon and nitrogen fertilization at the booting stage on non-structural carbohydrate content in stem sheath at maturity stage and soluble protein content in leaf at heading stage in fragrant rice. (**A**,**B**): non-structural carbohydrate content in 2021 and 2022; (**C**,**D**): soluble protein content in 2021 and 2022. Different letters represent significant differences between treatments within a variety. DW: dry weight. FW: fresh weight. For a description of the treatments, see Table 1.

The carbon and nitrogen application treatments resulted in higher non-structural carbohydrate content in the stem sheath in Meixiangzhan 2 and Xiangyaxiangzhan in 2022 (Figure 4A,B). Compared with the C0N0 treatment, the C0N1, C0N2, C1N2, and C2N1 treatments significantly increased the non-structural carbohydrate content in the stem sheath in Meixiangzhan 2 in 2021. The C0N1, C1N0, C1N2, C2N0, C2N1, and C2N2 treatments significantly increased the non-structural carbohydrate content in the stem sheath in Xiangyaxiangzhan in 2021. In a two-year study, C1N2 had the best lifting efficiency. The enhancement across most carbon and nitrogen application treatments in protein content was detected at various growth stages for both cultivars in both experimental years (Figure 4C,D). For Meixiangzhan 2, all carbon or nitrogen treatments increased the soluble protein content at the tassel stage, with C0N2 having the best effect.

### 3.5. Antioxidant Defense and Osmoregulation

**Figure 5:**
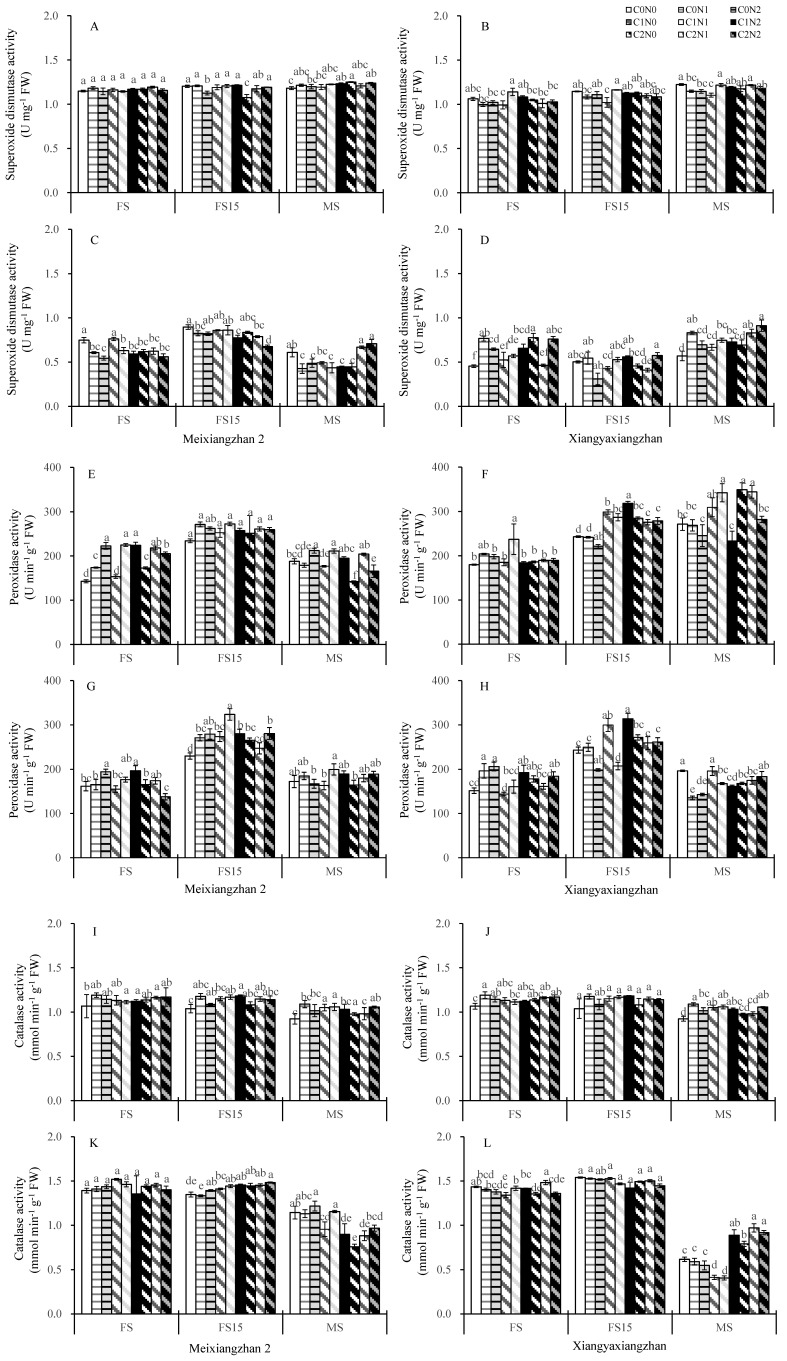
Effect of carbon and nitrogen fertilization at the booting stage on the activity of superoxide dismutase, peroxidase, and catalase in fragrant rice ((**A**,**B**,**E**,**F**,**I**,**J**) in 2021; (**C**,**D**,**G**,**H**,**K**,**L**) in 2022). Different letters represent significant differences between treatments within a variety. FS: heading stage, FS15: 15 days after heading, MS: maturity stage, FW: fresh weight. For a description of the treatments, see Table 1.

In 2021, the superoxide dismutase (SOD) activity of the two fragrant rice cultivars exhibited minor variations across treatments at different growth stages. In 2022, Xiangyaxiangzhan maintained higher SOD activity under carbon and nitrogen application treatments, whereas some treatments reduced SOD activity in Meixiangzhan 2 (Figure 5A–D). The peroxidase activity remained consistently elevated in both fragrant rice cultivars throughout the 2021 and 2022 growing seasons, with the notable exception of specific treatment groups observed in Xiangyaxiangzhan during the 2022 growing season (Figure 5E–H). The catalase activity demonstrated sustained enhancement across most carbon and nitrogen application treatments, maintaining higher levels through various growth stages for both cultivars in both experimental years (Figure 5I–L).

**Figure 6:**
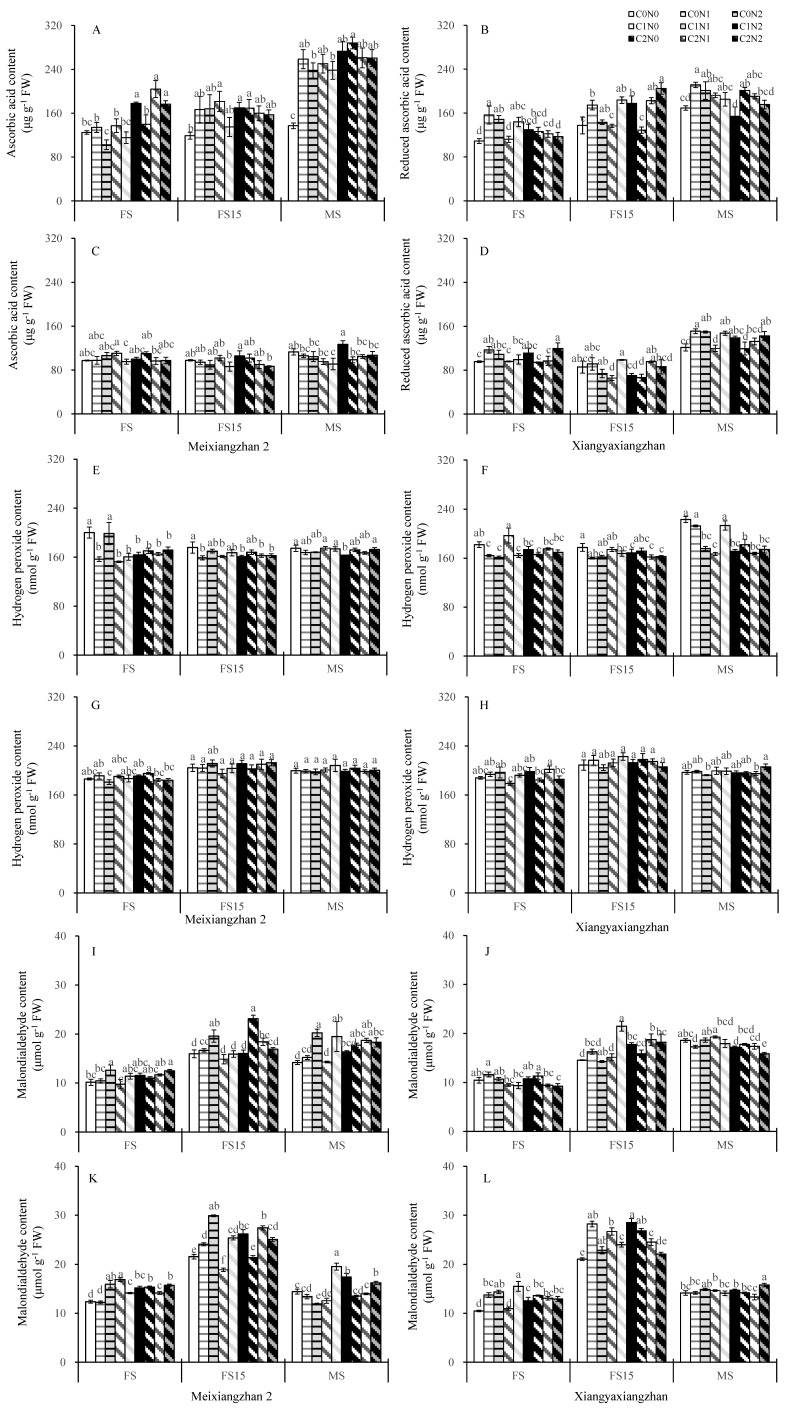
Effect of carbon and nitrogen fertilization at the booting stage on the content of ascorbic acid, hydrogen peroxide, and malondialdehyde in fragrant rice ((**A**,**B**,**E**,**F**,**I**,**J**) in 2021; (**C**,**D**,**G**,**H**,**K**,**L**) in 2022). Different letters represent significant differences between treatments within a variety. FS: heading stage, FS15: 15 days after heading, MS: maturity stage, FW: fresh weight. For a description of the treatments, see Table 1.

The ascorbic acid content was kept at a high level across most carbon and nitrogen application treatments at different growth stages for both cultivars in both experimental years. For Xiangyaxiangzhan, the anticyclic acid content was lower under the N0 treatments. For Meixiangzhan 2, only the C1N2 treatment had a higher anticyclic acid content than C0N0 at all stages (Figure 6A–D). Most carbon and nitrogen application treatments resulted in lower or slightly lower changes in the hydrogen peroxide content (Figure 6E–H). There were minor variations in the malondialdehyde content across treatments at different growth stages for both cultivars in both experimental years (Figure 6I–L). At 15 days after heading, a substantial increase in malondialdehyde content was observed in both varieties. In most cases, the malondialdehyde content decreased in Meixiangzhan 2 under the C1N0-treated, while it increased under the C0N2 treatment.

### 3.6. Correlation Analysis

**Figure 7:**
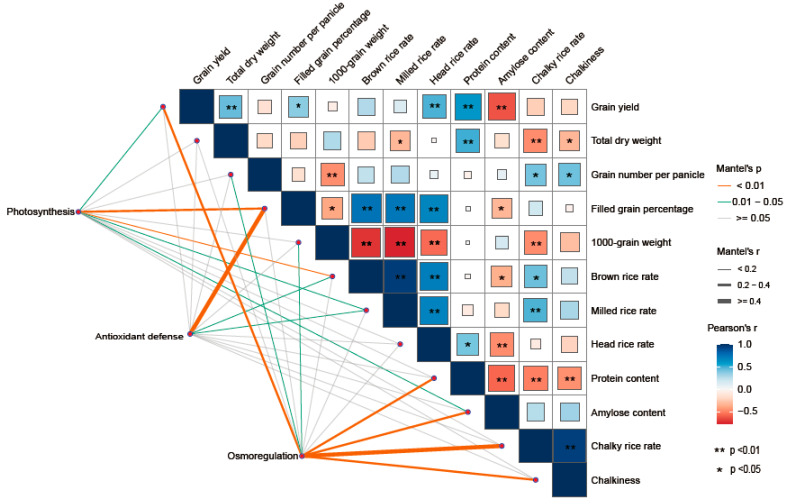
Correlation analysis of grain yield, grain quality, and the parameters of photosynthesis, antioxidant defense, and osmoregulation.

The correlation analysis indicated that the grain yield was positively correlated with the total dry weight, filled-grain percentage, head rice rate, protein content, photosynthetic rate at heading, and non-structural carbohydrate content and negatively correlated with the amylose content (*p* < 0.05). Milled quality (brown rice rate, milled rice rate, and head rice rate) was positively correlated with the filled-grain percentage, chalky rice rate, and negatively correlated with the 1000-grain weight and amylose content (*p* < 0.05). The protein content was positively correlated with total dry matter weight and head rice rate and negatively correlated with the amylose content, chalky rice rate, and chalkiness (*p* < 0.05). The analysis further demonstrated that grain yield is closely associated with parameters related to photosynthesis and osmoregulation. The grain number per panicle and 1000-grain weight exhibited a high correlation with characteristics linked to osmoregulation processes. The filled-grain percentage was suggested to be closely connected with parameters representing photosynthesis and antioxidant defense. The brown rice rate and milled rice rate were significantly related to the parameters of photosynthesis and antioxidant defense. Additionally, the protein content displayed a strong relationship with osmoregulation-related parameters, while the amylose content showed close associations with both photosynthesis and osmoregulation. Furthermore, chalky rice rate and chalkiness demonstrated significant correlations with osmoregulation-associated traits (Figure 7).

## 4. Discussion

Fragrant rice yield is regulated and can be increased with suitable carbon and nitrogen treatments. Urea has been shown to have a positive effect on yield in fragrant rice [53,54], and spraying glucose can alleviate the loss of rice yield due to drought stress [55]. In this study, both the carbon and nitrogen treatments significantly increased the yield of fragrant rice. At the same carbon level, the yield of fragrant rice increased with increasing nitrogen application, and the effect of nitrogen on yield improvement was stronger than that of the carbon treatments (Table 1), which is in agreement with the findings of Mo et al. [53]. In addition, the carbon and nitrogen interaction treatments were more effective in increasing yields. Fragrant rice sprayed with 150 mg L^−1^ glucose and 100 mg L^−1^ urea at the booting stage presented relatively high yields, but the difference between this treatment and other carbon and nitrogen interaction treatments was minor (Table 1). In previous studies, carbon or nitrogen treatments affected yield composition. Cong et al. reported that nitrogen fertilizer application increased the panicle per square meter, grain number per panicle, thousand grain weight, and filling grain percentage [56]. However, an integrative analysis by Xiao et al. revealed that nitrogen fertilizer application significantly increased the panicle per square meter and grain number per panicle while slightly decreasing the thousand grain weight and filling grain percentage [57]. Zhang et al. reported that spraying exogenous glucose resulted in a significant increase in panicle per square meter, grain number per panicle, and the thousand grain weight [55]. While studying the effects of high temperature on rice, Jiang et al. reported that sucrose alleviated high-temperature stress and increased the thousand-grain weight, which decreased in response to high temperature [31]. The results of this study revealed that the grain number per panicle and the filling grain percentage of fragrant rice were greater under the carbon and nitrogen interaction treatments than under the other treatments, indicating that the carbon treatments could mitigate the negative effects of nitrogen fertilizer on filling grain percentage and thus increase the yield of fragrant rice (Table 2, Figure 7). Among all the treatments, the application of 100 mg L^−1^ urea had a higher grain number per spike and filling rate, and the highest yield was obtained with the application of 150 mg L^−1^ glucose (Table 1). Furthermore, we found that the amylose content exhibited a highly significant positive correlation with grain yield, which is consistent with the findings of Ishimaru et al. [24]. In contrast, total dry weight, head rice percentage, and protein content were significantly and positively correlated with grain yield, and these results suggest that some of the carbohydrates are accumulated as starch in rice, and the others are used for plant growth. These results are consistent with the findings of Li et al. [22], further suggesting that altering the allocation of carbohydrates in rice can effectively promote yield increases. Additionally, treatments involving the interaction of carbon and nitrogen can encourage the flow of carbohydrates toward reproductive growth.

Rice quality is one of the most important indicators of the growth of fragrant rice, which is assessed with milling quality, nutritional quality, and appearance quality [58]. Ma et al. reported that rice cultivars of superior quality have lower grain protein content and lower amylose content than other cultivars [59]. Grain chalkiness is an adverse characteristic that negatively affects the appearance quality of rice [60]. Past studies have shown that rice quality significantly responds to carbon and nitrogen substances. The application of nitrogen fertilizer can improve milling quality [61], significantly increase the protein content, and reduce the amylose content of rice [62]. In addition, increasing the ratio of nitrogen in the spike fertilizer can reduce chalkiness and the chalky rice rate [63]. Rice quality is also regulated by carbon fertilization. The application of carbon fertilizer improves the taste of rice by reducing chalkiness and protein content [34], and lower amounts of biochar fertilizer (carbon fertilizer) are beneficial for improving the milled rice rate and appearance quality in rice [64]. In the present study, carbon and nitrogen fertilizer treatments significantly increased the protein content of fragrant rice, with C1N2 treatment showing the best performance in fragrant rice varieties and having the highest head rice rate in 2021 (Table 3). These findings indicated that the carbon and nitrogen interaction treatments suppressed the reduction in rice taste caused by the application of nitrogen fertilizer. Figure 7 shows that the protein content showed a highly significant negative correlation with straight-chain starch content, chalky rice percentage, and chalkiness, while the straight-chain starch content showed a positive correlation with chalky rice percentage and chalkiness. Chen’s research indicates that a higher amylose concentration leads to more translucent grains [65], which is consistent with our findings. In our study, the thousand-kernel weight showed highly significant negative correlations with brown rice rate, milling rate, and head rice rate, as well as negative correlations with the chalky rice rate and chalkiness. This suggests that as grain weight increases, it mainly increases the weight of the external structures of the rice kernel, such as hulls and other parts of the grain.

Higher dry matter accumulation is essential for the high yield and superior quality of rice [66]. Exogenous carbon and nitrogen substances affect the rate of carbon and nitrogen substance transport in crops and alter the accumulation of carbon and nitrogen substances in plants, which manifests as changes in the weight of dry matter [21,67]. In this study, the carbon and nitrogen treatments significantly increased the dry matter weights of the aboveground organs of fragrant rice, and the effect of the nitrogen treatment was particularly significant. The most significant increase in the dry matter of fragrant rice was observed under the C1 treatments (Table 1). Combining the performance of the two varieties, the sprayed treatments with 150 mg L^−1^ glucose and 100 mg L^−1^ urea (C1N2) had the most significant effect on dry matter weight, but the difference was minor compared to the other carbon and nitrogen interaction treatments. The increase in total dry weight of fragrant rice resulted from both enhanced leaf area index and increased tiller number. Although the carbon and nitrogen treatments induced an upward trend in leaf area index (Figure 2), the magnitude of this increase was considerably smaller than that of total dry weight. This discrepancy may be attributed to the fact that carbon and nitrogen treatments, particularly the C1N2 treatment, stimulated early growth and tillering in fragrant rice, ultimately leading to a substantial yield improvement. Yin et al. [68] and Liu et al. [69] verified the important role played by carbon or nitrogen substances in the accumulation of dry matter in crops. Guo et al. [33] reported that spraying appropriate concentrations of carbon and nitrogen substances could significantly increase the dry matter weight of rice. These findings suggest that carbon and nitrogen compounds treatments can increase the dry matter weight of fragrant rice and accumulate the necessary energy sources for shifting from nutritive to reproductive growth, thus contributing to its growth quality and yield. Photosynthesis is the main form of carbon metabolism in fragrant rice, the non-structural carbohydrates it produces are stored in the stem sheaths before heading, and their accumulation is positively correlated with dry matter accumulation; higher dry matter accumulation has been shown to contribute to the total photosynthetic intensity of the plant [70,71]. After heading, it becomes the main source of grain content, which is significantly associated with the formation of yield and quality [72]. In the present study, the application of N fertilizer significantly increased the non-structural carbohydrates of fragrant rice, and C1N2 significantly boosted the non-structural carbohydrates of fragrant rice leaves in a multi-year experiment involving two varieties (Figure 4). Exogenous carbon and nitrogen spraying can not only significantly increase the dry matter weight of crops and expand the capacity of storage organs (i.e., “sink”) but also effectively enhance their photosynthetic properties, thereby strengthening the production intensity of photosynthetic organs (i.e., “sources”). In this study, the effects of carbon and nitrogen treatments on photosynthetic characteristics of fragrant rice were investigated, and the results showed that the combined carbon and nitrogen treatments significantly increased the photosynthetic rate, leaf area index, and total chlorophyll content of fragrant rice leaves (Figure 2 and Figure 3). Among them, the effect of nitrogen treatment was more pronounced. The photosynthetic rate and leaf area index were significantly increased after the nitrogen-containing treatment, and the three treatments of C0N2, C1N1, and C1N2 had a promotional effect on the two fragrant rice cultivars, which is in line with the results of the study by Zhou and Yang [73]. Compared with the other treatments, the carbon and nitrogen interaction treatment had a greater photosynthetic intensity. The regulation of crop photosynthesis by exogenous glucose and nitrogen fertilizer has also been verified in previous studies [20,63]. Photosynthesis is the foundation of yield formation in rice, as robust productive organs simultaneously promote the formation of panicles and the production and distribution of dry matter [74], laying the groundwork for high yield in terms of both quantity and quality. Nitrogen (N), as an important environmental factor, influences the growth and development of rice, including tillering. Research has shown that nitrogen can act as a signaling factor, inducing the high expression of members of the nitrate transporter gene family, thereby promoting the number of panicles and the number of grains per panicle, ultimately increasing yield [75]. This aligns with the results of this study. Wang and Zhou demonstrated that nitrogen fertilizer could significantly enhance chlorophyll content, photosynthetic rate, peroxidase (POD), and superoxide dismutase (SOD) activities in plant leaves, while reducing the respiratory rate and increasing the soluble sugar content and soluble protein content in flag leaves, as well as root system vitality [76]. Zhou et al. revealed that carbon fertilizer could effectively improve the activities of POD, SOD, and catalase (CAT) in plant leaves under stress conditions, while also elevating soluble protein and soluble sugar contents [77]. Similar to the previously reported studies, in the present study, we were able to increase the soluble protein content in the leaves of fragrant rice to some extent by using carbon and nitrogen treatments (Figure 4C,D). Feng et al. found that foliar application of different carbon–nitrogen ratios significantly enhanced SOD, POD, and CAT activities in plant leaves, with reduced malondialdehyde (MDA) accumulation [78]. This study indicates that appropriate carbon–nitrogen treatments facilitate the improvement of stress-resistant physiological characteristics in rice plants (Figure 5 and Figure 6). Compared with the control, the treatment with applied N fertilizer significantly increased the POD content of fragrant rice at all periods; moreover, both CAT and ascorbic acid contents remained at high levels under appropriate carbon-to-nitrogen ratios, whereas the hydrogen peroxide content decreased, which is in agreement with the results of previous studies. Meanwhile, the antioxidant defense capacity was highly correlated with the full grain rate, brown rice rate, and milling rate (Figure 7), suggesting that the optimal carbon-to-nitrogen ratio could improve the antioxidant defense capacity of rice, which could indirectly improve the milling quality of rice and have a significant effect on yield.

Carbon and nitrogen metabolism is a crucial physiological process in the growth of fragrant rice, and its coordinated operation can increase the grain-filling rate and achieve high yield and superior quality [79]. Numerous studies have shown that the application of exogenous carbon and nitrogen substances can significantly affect the carbon and nitrogen transport rates in crops and disturb the dynamic balance of carbon and nitrogen metabolism [31,80,81]. However, the reestablished balance of carbon and nitrogen metabolism tends toward a more favorable direction for crop growth, demonstrating a positive regulatory effect [82,83]. In the present study, photosynthesis was positively correlated with milled grain percentage, protein content, and yield and negatively correlated with straight-chain starch content. In addition, dry matter showed highly significant positive correlations with both yield and protein and negative correlations with grain chalkiness and linear amylose content (Figure 7). These findings indicate that the carbon and nitrogen interaction treatments improved the yield, milling quality, protein content, and appearance quality of fragrant rice by increasing photosynthesis and dry matter, thereby reducing chalkiness and the amylose content. Carbonitriding treatment was significantly associated with yield, rice quality, and milling quality, and it may have significantly improved the efficiency of photosynthesis and carbon metabolism, thereby disturbing the original carbon and nitrogen metabolism equilibrium. Therefore, the new carbon and nitrogen balance increased the rate of carbon and nitrogen synthesis and transport in fragrant rice, significantly increased dry matter accumulation, provided sufficient nutrients for grain filling, and prolonged the time of carbon and nitrogen transport to the grains, which resulted in a relatively high yield and better quality of fragrant rice. However, further research is needed to validate other aspects of this concept, such as nitrogen metabolism.

## 5. Conclusions

In summary, spraying carbon and nitrogen fertilizers at the tasseling stage of rice growth can effectively improve its carbon and nitrogen utilization efficiency and promote the rational allocation of nutrients by optimizing the balance of carbon and nitrogen metabolism and increasing the production capacity of the source and the holding capacity of the reservoir. In this study, the spraying of 150 mg L^−1^ glucose and 100 mg L^−1^ urea at the tasseling stage of fragrant rice growth can realize the improvement of yield and rice quality by enhancing the photosynthetic intensity, antioxidant defense ability, and osmotic adjustment ability of fragrant rice.

## Figures and Tables

**Table 1:** Effect of carbon and nitrogen fertilization at the booting stage on grain yield and total dry weight in fragrant rice.

		Grain Yield	(t hm^−2^)	Total Dry Weight	(t hm^−2^)
Cultivar	Treatment	2021	2022	2021	2022
Meixiangzhan 2	C0N0	3.57 b	5.26 d	6.29 d	10.04 e
	C0N1	4.23 ab	6.24 c	8.77 cd	14.67 abcd
	C0N2	4.30 ab	6.38 bc	9.33 bc	11.8 cde
	C1N0	4.34 ab	5.98 c	13.13 a	15.42 abc
	C1N1	4.88 ab	6.40 bc	10.05 bc	15.74 ab
	C1N2	5.28 ab	7.22 a	10.77 abc	16.15 a
	C2N0	3.83 b	6.17 c	10.19 bc	14.36 abcd
	C2N1	6.07 a	6.89 ab	11.76 ab	12.31 bcde
	C2N2	5.01 ab	6.98 ab	11.6 ab	11.64 de
Xiangyaxiangzhan	C0N0	4.30 a	4.24 e	11.22 e	10.20 d
	C0N1	4.48 a	5.21 c	12.32 cde	15.09 ab
	C0N2	4.42 a	5.69 bc	14.49 b	13.43 abc
	C1N0	4.63 a	4.71 de	12.20 de	14.11 abc
	C1N1	4.50 a	5.56 bc	17.95 a	16.14 a
	C1N2	4.82 a	6.58 a	14.29 bc	14.01 abc
	C2N0	4.63 a	5.20 cd	13.93 bcd	12.12 bcd
	C2N1	4.76 a	6.51 a	14.95 b	15.27 a
	C2N2	4.50 a	5.72 b	11.54 e	11.31 cd
ANOVA	Y	**		ns	
	C	**		**	
	T	**		**	
	Y × C	**		**	
	Y × T	**		ns	
	C × T	ns		**	
	Y × C × T	*		ns	

Values with different small letters in the same line are significant differences. Y, C, and T represent year, cultivar, and treatment of spraying glucose and urea, respectively; ns indicates non-significance, and * and ** indicate significance at *p* < 0.05 and *p* < 0.01, respectively. C0: 0 mg L^−1^ glucose, C1: 150 mg L^−1^ glucose, C2: 300 mg L^−1^ glucose, N0: 0 mg L^−1^ urea, N1: 50 mg L^−1^ urea, N2: 100 mg L^−1^ urea.

**Table 2:** Effect of carbon and nitrogen fertilization at the booting stage on yield components in fragrant rice.

Cultivar	Treatment	Grain Number per Panicle		Filled-Grain Percentage (%)		1000-Grain Weight (g)	
		2021	2022	2021	2022	2021	2022
Meixiangzhan 2	C0N0	132.25 abc	126.18 abc	83.84 a	81.61 ab	17.66 bc	18.09 a
	C0N1	132.72 abc	114.11 c	76.10 abcd	86.33 a	17.45 c	17.88 ab
	C0N2	147.92 a	141.12 ab	80.68 abc	86.25 a	18.19 ab	17.71 abc
	C1N0	125.97 bc	116.15 bc	78.89 abc	78.73 bc	17.41 c	17.47 bc
	C1N1	131.77 abc	118.96 bc	67.31 d	81.26 ab	17.52 c	18.14 a
	C1N2	139.28 ab	147.32 a	73.51 bcd	79.95 abc	17.68 bc	18.12 a
	C2N0	116.03 c	126.96 abc	82.82 ab	81.70 ab	18.58 a	17.70 abc
	C2N1	126.00 bc	127.99 abc	81.58 abc	76.77 bc	17.68 bc	17.31 c
	C2N2	139.73 ab	132.29 abc	72.63 cd	73.56 c	17.64 bc	17.64 abc
Xiangyaxiangzhan	C0N0	144.90 a	108.56 cd	52.81 bc	66.92 a	18.35 a	19.14 a
	C0N1	130.30 a	126.25 a	49.15 c	69.33 a	17.96 a	19.13 a
	C0N2	144.33 a	113.59 abcd	56.20 bc	64.93 a	18.41 a	19.05 a
	C1N0	139.47 a	103.17 d	54.18 bc	71.04 a	18.21 a	19.14 a
	C1N1	133.63 a	112.20 bcd	53.09 bc	66.08 a	18.26 a	19.09 a
	C1N2	127.27 a	124.69 ab	69.10 a	68.33 a	18.35 a	19.14 a
	C2N0	130.82 a	112.75 abcd	52.80 bc	64.29 a	18.37 a	19.30 a
	C2N1	141.48 a	105.12 d	58.02 b	68.46 a	18.54 a	19.06 a
	C2N2	137.85 a	118.75 abc	60.11 b	68.38 a	18.41 a	19.24 a
ANOVA	Y	**		**		**	
	C	ns		**		**	
	T	**		ns		ns	
	Y × C	*		**		**	
	Y × T	ns		*		ns	
	C × T	ns		**		ns	
	Y × C × T	ns		*		ns	

Values with different small letters in the same line are significant differences. Y, C, and T represent year, cultivar, and treatment of spraying glucose and urea, respectively; ns indicates non-significance, and * and ** indicate significance at *p* < 0.05 and *p* < 0.01, respectively. For a description of the treatments, see Table 1.

**Table 3:** Effect of carbon and nitrogen fertilization at the booting stage on grain quality in fragrant rice.

Year/Cultivar	Treatment	Brown Rice Rate (%)	Milled Rice Rate (%)	Head Rice Rate (%)	Protein Content (%)	Amylose Content (%)	Chalky Rice Rate (%)	Chalkiness (%)
2021								
Meixiangzhan 2	C0N0	74.10 a	66.07 a	57.82 a	7.68 d	18.80 abc	14.29 a	3.56 b
	C0N1	74.83 a	66.85 a	54.03 a	7.78 cd	19.05 ab	21.77 a	6.91 a
	C0N2	75.73 a	67.41 a	49.51 a	7.85 c	18.35 cd	17.94 a	5.32 ab
	C1N0	74.19 a	67.02 a	53.10 a	7.83 c	19.10 a	18.02 a	5.40 ab
	C1N1	74.83 a	66.45 a	53.88 a	7.98 ab	18.58 bc	17.41 a	4.95 ab
	C1N2	75.69 a	67.54 a	56.85 a	8.03 a	18.39 cd	21.22 a	6.51 a
	C2N0	74.72 a	66.75 a	50.36 a	7.68 d	18.90 ab	22.23 a	6.67 ab
	C2N1	74.91 a	66.40 a	50.38 a	7.85 c	18.95 ab	18.67 a	5.27 ab
	C2N2	75.02 a	66.92 a	51.57 a	7.88 bc	17.93 d	20.42 a	6.01 ab
Xiangyaxiangzhan	C0N0	71.22 a	61.41 ab	49.73 ab	8.23 abc	18.40 c	13.79 a	4.67 a
	C0N1	71.89 a	62.39 ab	48.93 bc	8.15 bc	18.55 bc	14.79 a	5.53 a
	C0N2	72.23 a	64.97 a	51.62 ab	8.13 c	19.00 a	14.88 a	5.28 a
	C1N0	69.94 a	59.93 b	44.63 c	8.15 bc	18.63 abc	18.02 a	5.40 a
	C1N1	71.21 a	62.32 ab	52.71 ab	8.28 ab	18.75 abc	9.68 a	3.01 a
	C1N2	72.47 a	63.75 a	54.39 a	8.33 a	18.85 ab	17.16 a	5.86 a
	C2N0	71.62 a	62.77 ab	48.70 bc	8.20 abc	18.88 ab	11.23 a	3.53 a
	C2N1	72.88 a	63.87 a	48.80 bc	7.90 d	18.80 abc	14.63 a	5.32 a
	C2N2	72.15 a	63.43 ab	53.60 ab	7.85 d	18.85 ab	12.73 a	3.79 a
2022								
Meixiangzhan 2	C0N0	74.76 b	67.26 a	59.32 a	8.60 a	18.10 ab	12.59 ab	3.41 ab
	C0N1	75.07 ab	66.80 a	60.14 a	8.33 cd	18.25 a	10.51 ab	2.71 b
	C0N2	73.50 c	65.75 a	58.55 a	8.45 b	18.13 ab	10.73 ab	3.43 ab
	C1N0	75.66 ab	66.83 a	59.07 a	8.38 bc	17.85 b	10.87 ab	2.83 b
	C1N1	75.70 a	66.06 a	59.81 a	8.33 cd	18.15 ab	9.70 b	2.82 b
	C1N2	75.18 ab	65.44 a	59.13 a	8.63 a	18.08 ab	13.17 ab	4.11 ab
	C2N0	75.61 ab	65.64 a	57.95 a	8.25 de	18.05 ab	16.84 a	4.73 ab
	C2N1	75.25 ab	66.80 a	59.71 a	8.28 d	18.10 ab	15.77 ab	5.27 a
	C2N2	75.40 ab	66.63 a	58.45 a	8.18 e	18.03 ab	16.98 a	5.51 a
Xiangyaxiangzhan	C0N0	72.30 ab	62.74 a	49.35 cd	8.25 a	18.48 ab	10.45 a	2.98 a
	C0N1	72.69 a	62.14 ab	50.30 bc	7.95 c	18.48 ab	12.49 a	4.14 a
	C0N2	71.22 bcd	61.64 ab	48.37 d	8.13 b	18.33 b	11.28 a	3.62 a
	C1N0	70.43 d	61.24 b	51.49 ab	8.00 c	18.50 ab	8.43 a	2.60 a
	C1N1	70.86 cd	61.95 ab	48.28 d	8.25 a	18.40 ab	14.04 a	4.64 a
	C1N2	71.45 bcd	62.05 ab	50.18 bc	8.20 ab	18.58 ab	12.83 a	4.37 a
	C2N0	71.98 abc	61.58 ab	50.26 bc	8.00 c	18.43 ab	10.99 a	3.93 a
	C2N1	71.44 bcd	62.06 ab	50.50 bc	8.20 ab	18.38 ab	11.57 a	4.12 a
	C2N2	71.33 bcd	62.55 ab	53.17 a	8.03 c	18.68 a	13.29 a	4.41 a
ANOVA								
Y		ns	ns	ns	**	**	*	*
C		**	**	**	ns	**	**	ns
T		ns	ns	ns	**	ns	ns	ns
Y × C		ns	ns	*	**	**	*	ns
Y × T		ns	ns	ns	**	*	ns	ns
C × T		ns	ns	ns	**	**	ns	ns
Y × C × T		ns	ns	ns	**	**	ns	ns

Values with different small letters in the same line are significant differences. Y, C, and T represent year, cultivar, and treatment of spraying glucose and urea, respectively; ns indicates non-significance, and * and ** indicate significance at *p* < 0.05 and *p* < 0.01, respectively. For a description of the treatments, see Table 1.

## Data Availability

Data will be made available on request.
